# FDUNet-LRSA: efficient frequency-adaptive segmentation for automated crop disease inspection

**DOI:** 10.3389/fpls.2026.1963106

**Published:** 2026-10-16

**Authors:** Ru Han, Lei Shu, Grzegorz Cielniak, Yan Zhu, Jucheng Yang, Fan Yang, Weihan Song, Qian Zhang, Kai Huang

**Affiliations:** 1 College of Smart Agriculture (College of Artificial Intelligence), Nanjing Agricultural University, Nanjing, China; 2 School of Engineering and Physical Sciences, University of Lincoln, Lincoln, United Kingdom; 3 Lincoln Institute for Agri-food Technology, University of Lincoln, Lincoln, United Kingdom; 4 Guangxi Artificial Intelligence Academic and Guilin University of Electronic Technology Nanning Research Institute, Guangxi, China; 5 School of Electrical Engineering and Automation, Jiangsu Normal University, Xuzhou, China; 6 Key Laboratory of Sweetpotato Biology and Genetic Breeding, Ministry of Agriculture and Rural Affairs, Xuzhou Institute of Agricultural Sciences in Jiangsu Xuhuai District, Xuzhou, China

**Keywords:** agricultural automation, computer vision, frequency-domain modeling, semantic segmentation, visual inspection

## Abstract

The transition toward industrialized and automated agriculture requires reliable machine vision for high-throughput crop health monitoring and field decision support. Automated sweetpotato disease inspection, however, remains challenging because field images exhibit substantial variations in leaf scale, appearance, illumination, occlusion, and background complexity. Conventional U-shaped networks rely primarily on fixed convolutional kernels and local feature aggregation, whereas Transformer-based alternatives often introduce considerable computational overhead. This article proposes FDUNet-LRSA, a frequency-adaptive segmentation network that integrates frequency-domain dynamic convolution, low-resolution self-attention, and boundary-aware refinement. Frequency-domain dynamic convolution reconstructs input-dependent spatial kernels to improve feature adaptation under changing imaging conditions. Low-resolution self-attention captures long-range contextual dependencies in a fixed compact feature space, while boundary-aware refinement preserves irregular leaf contours during mask reconstruction. Under the reported evaluation protocol, FDUNet-LRSA achieved a mean test foreground IoU of 0.94 and a Dice coefficient of 0.97, with 3.72 GFLOPs. Additional experiments indicate that task-oriented moderate augmentation improves generalization compared with stronger augmentation. The proposed network provides an efficient visual perception approach for automated crop inspection systems.

## 1 Introduction

Transition from labor-intensive farming toward industrialized and automated agricultural production is increasing the demand for reliable visual perception systems. In modern field operations, machine vision is increasingly integrated with handheld sensing devices, mobile agricultural machinery, robotic platforms, and edge-computing units to support high-throughput crop monitoring and data-driven management. Accurate crop-region segmentation is a fundamental perception capability in such systems because it directly affects subsequent disease assessment, plant-level analysis, and automated operational decisions.

Sweet potato virus disease (SPVD) is a major constraint on sweetpotato production and can cause substantial degradation in plant growth and crop quality (George et al., 2025). In field management, accurately extracting symptomatic leaves from complex canopy backgrounds is an important prerequisite for automated disease inspection, plant-level monitoring, and subsequent severity analysis. However, images acquired using handheld or mobile agricultural sensing platforms often exhibit substantial variations in viewing distance, camera angle, illumination, leaf orientation, and occlusion (Lai et al., 2024; Zhang et al., 2025b). These variations produce large changes in leaf scale and appearance, making reliable pixel-level segmentation difficult under practical field conditions.

Convolutional encoder–decoder networks, particularly U-shaped architectures, are widely used for agricultural image segmentation because skip connections help preserve spatial details while extracting semantic features (Das et al., 2025). However, fixed convolutional kernels have limited adaptability to scale and texture variations, and their local receptive fields restrict long-range context modeling. Transformer-based methods improve global-context representation but introduce substantial computational and memory overhead, while repeated downsampling and upsampling may further blur irregular leaf boundaries (Li and Zhou, 2025; Zhang et al., 2025a).

Existing studies have separately demonstrated the effectiveness of dynamic convolution, frequency-domain modeling, efficient self-attention, and boundary-aware refinement in general vision and segmentation tasks (Zhang et al., 2021, 2024). Therefore, this work does not claim the first introduction of these underlying techniques. However, their complementary roles have not been systematically investigated in field-oriented agricultural disease segmentation, where scale and appearance variations, discontinuous disease features, irregular leaf boundaries, and computational constraints must be addressed simultaneously.

To address this gap, this article proposes FDUNet-LRSA, a frequency-adaptive U-shaped network for segmenting SPVD-affected leaves. To the best of our knowledge, this is the first systematic exploration and verification of a unified local–global–boundary framework that coordinates frequency-adaptive local feature extraction, low-resolution global-context modeling, and boundary-aware refinement for agricultural disease segmentation (Pandey and Mishra, 2024; Ding et al., 2025). The first-time contribution therefore lies in the task-oriented coordination and systematic experimental verification of these complementary designs, rather than in the isolated introduction of dynamic convolution, frequency representation, self-attention, or Laplacian operators.

The proposed network further introduces several task-specific improvements over these existing technologies. First, frequency-domain dynamic convolution is embedded into hybrid convolutional blocks to reconstruct input-dependent spatial kernels while retaining the local inductive bias and efficiency of conventional convolution. Second, low-resolution self-attention is selectively placed only at deeper encoder, bottleneck, and decoder stages, allowing long-range dependencies to be modeled in a fixed *p*×*p* feature space without the cost of full-resolution attention (Goyal et al., 2024; Ren et al., 2025; Malik and Mahmud, 2026). Third, conventional Laplacian boundary extraction is extended with a learnable attention gate and residual modulation, thereby reinforcing reliable disease boundaries while suppressing noise caused by complex field backgrounds.

The main contributions of this work are summarized as follows:

1. First systematic task-level investigation: this study is the first to systematically explore and verify the coordinated use of frequency-adaptive local modeling, efficient global-context aggregation, and boundary refinement within a unified agricultural disease segmentation framework
2. Task-oriented improvements over existing techniques: an FDConv-based hybrid convolutional design and selectively placed LRSA blocks are developed to improve adaptability to leaf-scale and appearance variations while reducing the computational cost of global-context modeling.
3. Boundary refinement and systematic validation: a gated Laplacian boundary module is introduced to enhance irregular leaf contours while suppressing noisy edge responses. Progressive ablation experiments, comparisons with representative segmentation networks, complexity analysis, and controlled augmentation experiments verify the individual and combined effects of these designs.

## 2 Related works

The related literature is reviewed from three complementary perspectives corresponding to the principal challenges addressed in this work. First, agricultural image segmentation is examined to summarize the development of field-oriented encoder–decoder and Transformer-based frameworks. Second, dynamic convolution and frequency-domain modeling are reviewed because they provide the technical basis for input-adaptive local feature extraction. Third, efficient attention and boundary refinement are discussed to clarify existing approaches for global-context aggregation and contour preservation. This organization establishes the relationship between field segmentation requirements and the three principal components of FDUNet-LRSA.

### 2.1 Agricultural image segmentation

Deep learning-based semantic segmentation has been increasingly applied to automated crop monitoring, including leaf extraction, disease-region analysis, and field-scene understanding (Polly and Devi, 2024; Mehdipour et al., 2025). Encoder–decoder networks, particularly U-shaped architectures, remain widely used because skip connections combine high-level semantic information with fine spatial details. Attention mechanisms and multiscale context modules have further improved foreground localization under complex backgrounds. However, conventional convolutional networks primarily rely on fixed kernels and local feature aggregation, making them sensitive to changes in object scale, illumination, occlusion, and canopy structure (Wang et al., 2025).

Transformer-based architectures provide stronger long-range dependency modeling and have shown promising performance in agricultural and biomedical segmentation. Nevertheless, full-resolution or window-based attention generally increases computation, memory consumption, and training complexity. These limitations are particularly relevant to agricultural inspection systems, where image processing may need to be performed on resource-constrained computing platforms (Acharya et al., 2025). Therefore, an effective field segmentation model should preserve the spatial-detail advantages of U-shaped networks while incorporating global context at a manageable computational cost.

Visual–language fusion has also been investigated for agricultural disease segmentation (Hu et al., 2024). proposed LVF, which integrates image and text information for tomato disease segmentation. This work provides a multimodal perspective on agricultural disease analysis, complementing the present investigation of adaptive feature extraction and contextual modeling from RGB imagery.

### 2.2 Dynamic convolution and frequency-domain modeling

Dynamic convolution improves feature adaptability by generating input-dependent kernels or aggregating multiple candidate kernels according to image content. Compared with static convolution (Tian et al., 2021), this mechanism can better accommodate variations in object appearance and scale. Existing dynamic convolution methods, however, are commonly constructed directly in the spatial domain, and their adaptive kernel generation may introduce additional parameters or inference overhead.

Frequency-domain modeling provides an alternative means of representing convolutional filters and visual features. Spatial-frequency components encode texture transitions, contour changes, and structural variations that may be difficult to capture using fixed local operators alone. Recent studies have employed Fourier- or wavelet-based representations to enhance feature extraction efficiency and robustness. However, most existing approaches use frequency information for feature transformation or domain adaptation rather than for input-conditioned convolutional kernel generation (Mehta et al., 2023; Xiang et al., 2025; Xu et al., 2025). In this work, learnable frequency-domain kernel representations are combined with input-dependent weighting to reconstruct adaptive spatial kernels, enabling local feature extraction to respond to variations in leaf scale and appearance.

### 2.3 Efficient attention and boundary refinement

Self-attention is effective for modeling long-range semantic relationships but has quadratic complexity with respect to the number of spatial tokens when applied at full resolution (Jiao et al., 2022). Efficient attention mechanisms reduce this burden through token reduction, spatial pooling, sparse interaction, or restricted attention regions. Low-resolution self-attention performs global interaction in a fixed, compact feature space and then projects the contextual information back to the original resolution (Wu et al., 2023). This strategy provides global-context modeling with substantially lower computational demand than full-resolution attention.

Accurate agricultural segmentation also depends on preserving irregular contours, narrow leaf structures, and partially occluded boundaries. Standard encoder–decoder networks may produce over-smoothed masks because repeated resolution reduction weakens high-frequency edge information. Boundary-aware methods address this problem through auxiliary edge supervision, gradient operators, or attention-guided feature refinement. Nevertheless, global-context modeling and boundary enhancement are often studied independently. FDUNet-LRSA combines selectively placed low-resolution attention with Laplacian-guided boundary refinement, allowing global semantic consistency and local contour preservation to be jointly considered within a frequency-adaptive U-shaped architecture (Deng et al., 2023; Liu et al., 2025).

## 3 Proposed method

### 3.1 Overall architecture

This study performs whole-leaf foreground segmentation for sweetpotato virus disease inspection. The reference masks include both symptomatic and unaffected tissue within the annotated leaf, while pixels outside the leaf are labeled as background. Individual lesions are not annotated. Therefore, the predicted masks represent leaf extent rather than disease status, and the reported IoU and Dice scores evaluate whole-leaf segmentation, not lesion localization or disease diagnosis.

The proposed FDUNet-LRSA follows a symmetric encoder–decoder architecture for pixel-level segmentation of SPVD-affected leaves. As illustrated in **Figure 1**, the network consists of four encoder stages, a bottleneck, four decoder stages, and a boundary-aware output head. Frequency-domain dynamic convolution (FDConv) is used throughout the encoder to provide input-adaptive local feature extraction, whereas low-resolution self-attention (LRSA) is inserted at deeper stages to capture global contextual dependencies at reduced spatial resolution. The decoder progressively restores spatial resolution through bilinear upsampling and skip-feature fusion. A boundary-aware refinement module is applied before the final classifier to enhance irregular leaf contours. The detailed stage configurations, feature resolutions, channel dimensions, and principal components are summarized in **Table 1**.

**Figure 1:**
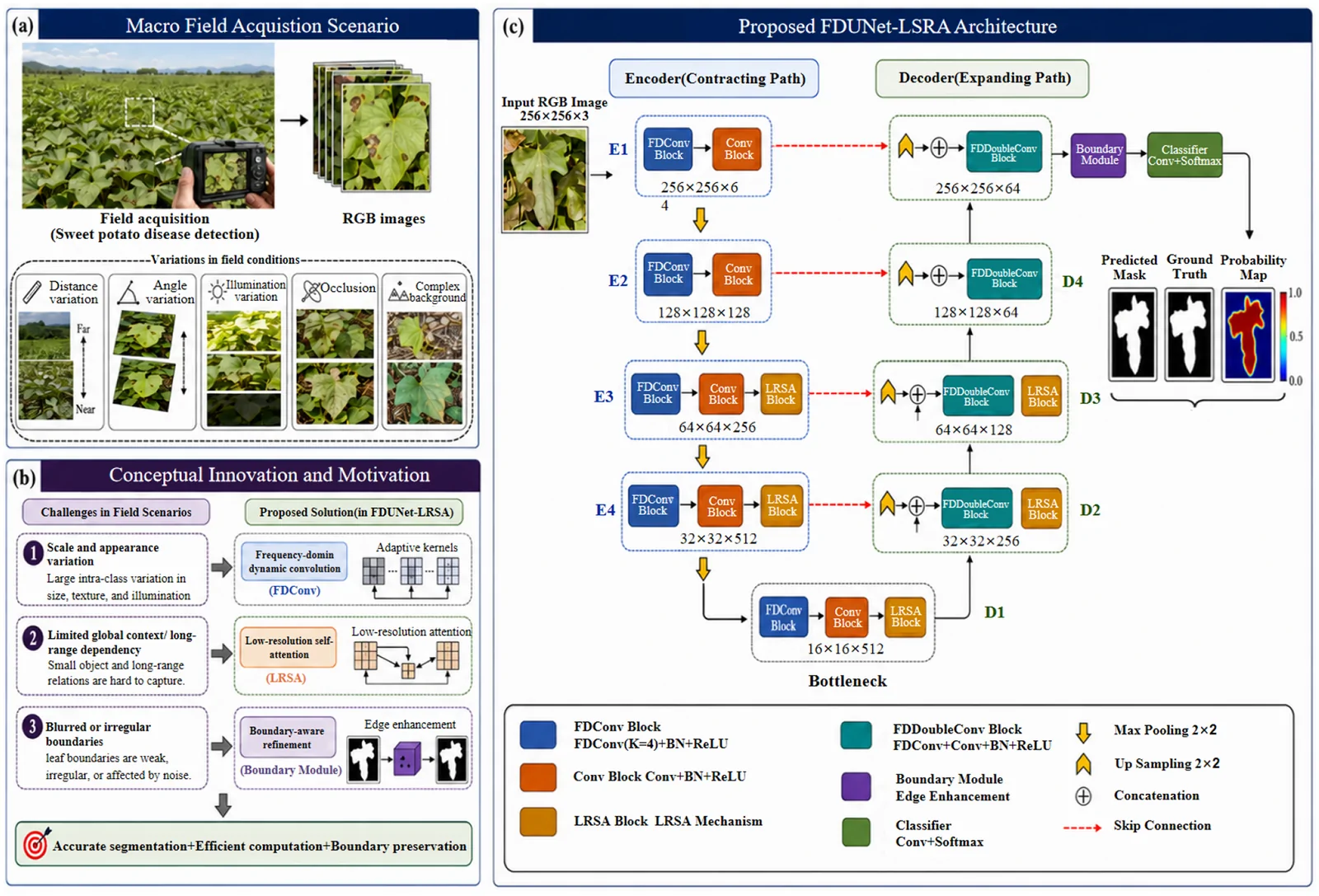
The overall architecture of FDUNet-LRSA. The network incorporates FDConv for input-adaptive feature extraction, LRSA for long-range dependency modeling at fixed low-resolution space, and a Boundary Module for edge enhancement.

**Table 1:** Structure of the FDUNet-LRSA network model.

Stage	Layer	Kernel/stride	Output size	Components
Encoder (contracting path)				
Encoder 1	FDConv1	3×3, 1	256×256×64	FDConv(*K* = 4) + BN + ReLU
	Conv2	3×3, 1	256×256×64	Conv + BN + ReLU
	MaxPooling	2×2, 2	128×128×64	–
Encoder 2	FDConv3	3×3, 1	128×128×128	FDConv(*K* = 4) + BN + ReLU
	Conv4	3×3, 1	128×128×128	Conv + BN + ReLU
	MaxPooling	2×2, 2	64×64×128	–
Encoder 3	FDConv5	3×3, 1	64×64×256	FDConv(*K* = 4) + BN + ReLU
	Conv6	3×3, 1	64×64×256	Conv + BN + ReLU
	LRSA Block	*p* = 7	64×64×256	LRSA(heads=4) + LFF
	MaxPooling	2×2, 2	32×32×256	–
Encoder 4	FDConv7	3×3, 1	32×32×512	FDConv(*K* = 4) + BN + ReLU
	Conv8	3×3, 1	32×32×512	Conv + BN + ReLU
	LRSA Block	*p* = 7	32×32×512	LRSA(heads=8) + LFF
	MaxPooling	2×2, 2	16×16×512	–
Bottleneck	FDConv9	3×3, 1	16×16×512	FDConv(*K* = 4) + BN + ReLU
	Conv10	3×3, 1	16×16×512	Conv + BN + ReLU
	LRSA Block	*p* = 7	16×16×512	LRSA(heads=8) + LFF
Decoder (expanding path)				
Decoder 1	Upsampling	×2	32×32×512	Bilinear Interpolation
	Concat	–	32×32×1024	Skip Connection
	FDDoubleConv	3×3, 1	32×32×256	FDConv + Conv + BN + ReLU
	LRSA Block	*p* = 7	32×32×256	LRSA(heads=8) + LFF
Decoder 2	Upsampling	×2	64×64×256	Bilinear Interpolation
	Concat	–	64×64×512	Skip Connection
	FDDoubleConv	3×3, 1	64×64×128	FDConv + Conv + BN + ReLU
	LRSA Block	*p* = 7	64×64×128	LRSA(heads=4) + LFF
Decoder 3	Upsampling	×2	128×128×128	Bilinear Interpolation
	Concat	–	128×128×256	Skip Connection
	FDDoubleConv	3×3, 1	128×128×64	FDConv + Conv + BN + ReLU
Decoder 4	Upsampling	×2	256×256×64	Bilinear Interpolation
	Concat	–	256×256×128	Skip Connection
	FDDoubleConv	3×3, 1	256×256×64	FDConv + Conv + BN + ReLU
Output				
Output	Boundary Module	3×3, 1	256×256×64	Edge Enhancement
	Classifier	1×1, 1	256×256×2	Conv + Softmax

*K* denotes the number of candidate kernels in FDConv; *p* denotes the pooled size in LRSA; LFF, Locality Feed-Forward.

Given an input image $I\in {\mathbb{R}}^{3\times H\times W}$, the encoder generates multiscale features ${\left\{E_{l}\right\}}_{l=1}^{4}$, where the spatial resolution is reduced by a factor of two at each stage. The first two encoder stages employ FDConv followed by standard convolution, whereas the deeper stages additionally incorporate LRSA. At the bottleneck, FDConv, standard convolution, and LRSA are combined to learn compact global–local representations.

In the decoder, each feature map is bilinearly upsampled and concatenated with the corresponding encoder feature through a skip connection. The fused feature is then refined using an FDDoubleConv block, consisting of FDConv followed by standard convolution. LRSA is retained in the two deeper decoder stages to preserve long-range semantic consistency during feature reconstruction. Finally, the boundary-aware module enhances contour-sensitive responses, and a 1×1 convolution followed by Softmax produces the two-channel segmentation output.

### 3.2 Frequency-domain dynamic convolution

Input-dependent kernel generation is an established strategy in dynamic convolution. CondConv aggregates expert kernels using sample-dependent coefficients, while ODConv extends dynamic modulation to multiple dimensions of the kernel space. In the formulation used here, the distinguishing implementation choice is to learn Fourier coefficients and reconstruct real-valued spatial kernels before convolution.

For an unconstrained kernel represented by a complete valid spectrum, Fourier parameterization does not by itself enlarge the set of representable spatial filters. Its effect on learning and segmentation performance must therefore be established empirically. We evaluate this effect using a spatial-domain dynamic convolution control with the same kernel size, number of candidate kernels, routing network, and insertion positions.

Conventional convolution applies a fixed spatial kernel to all input samples, which limits its adaptability to changes in leaf scale, texture, and imaging conditions. FDConv addresses this limitation by reconstructing sample-specific spatial kernels from a learnable frequency-domain kernel bank. The complete computational pipeline and tensor transformations of FDConv are summarized in **Table 2**.

**Table 2:** Internal structure of FDConv (frequency domain dynamic convolution) module.

Component	Operation	Output shape
Attention branch (dynamic weight generation)		
Input	Feature map ***X***	(*B*, *C_in_*, *H*, *w*)
GAP	AdaptiveAvgPool2d(1)	(*B*, *C_in_*, 1, 1)
Flatten	Reshape	(*B*, *C_in_*)
FC1	Linear(*C_in_*, *C_in_*/4)	(*B*, *C_in_*/4)
Activation	ReLU	(*B*, *C_in_*/4)
FC2	Linear(*C_in_*/4, K)	(*B*, *K*)
Attention	Softmax	(*B*, *K*)
Frequency domain kernel synthesis		
DFT Params	Learnable $W^{freq}\in \mathbb{C}$	(*K*, *N_freq_*, 2)
Weighting	$\overset{K}{\underset{i=1}{\sum }}\alpha_{i}W_{i}^{freq}$	(*B*, *N_freq_*, 2)
iRFFT2	Inverse Real FFT 2D	(*B*, *C_out_* · *k*, *C_in_* · *k*)
Reshape	Kernel formation	(*B*, *C_out_*, *C_in_*, *k*, *k*)
Dynamic convolution		
GroupConv	$X*W_{dynamic}$	(*B*, *C_out_*, *H*′, *W*′)

*K*, number of candidate kernels (default 4); *k*, kernel size (default 3); τ, temperature for attention softening; *N_freq_*, number of frequency components.

Bold values denote the relatively better results in the comparison.

Unlike conventional convolution where the same kernel parameters are shared across all input samples, field-acquired crop images usually exhibit large variations caused by illumination changes, leaf orientation, symptom severity, and background interference. Therefore, a fixed spatial kernel may not provide sufficient adaptability for extracting discriminative disease-related features. FDConv introduces an input-conditioned kernel generation mechanism, where the contribution of multiple candidate kernels is dynamically adjusted according to the global characteristics of the input feature map.

Specifically, the global descriptor summarizes the overall semantic response of the current feature representation, which enables the network to estimate the importance of different frequency components. This adaptive weighting strategy allows FDConv to emphasize informative patterns while suppressing irrelevant responses caused by environmental noise.

Let $X\in {\mathbb{R}}^{B\times C_{\text{in}}\times H\times W}$ denote an input feature map. A global descriptor is first obtained using global average pooling, and a lightweight gating network generates the sample-dependent kernel coefficients as shown Equation (1):

(1) $$\alpha =\text{Softmax}\left(\frac{W_{2}\delta \left(W_{1}\text{GAP}\left(X\right)\right)}{\tau }\right),$$

where *δ*(·) denotes ReLU, *τ* is a temperature coefficient, and $\alpha =\left[\alpha_{1},\ldots ,\alpha_{K}\right]$ contains the normalized weights for *K* candidate kernels.

As Equation (2) each candidate kernel is parameterized in the frequency domain as

(2) $${\hat{W}}_{i}\in {\mathbb{C}}^{C_{\text{out}}\times C_{\text{in}}\times k\times \left(\left[k/2\right]+1\right)}.$$

Instead of directly learning spatial kernels, FDConv represents each candidate kernel in the frequency domain. The motivation is that different frequency components correspond to different visual characteristics. Low-frequency components mainly preserve global structural information, such as leaf shape and large symptom regions, whereas high-frequency components capture local details including lesion boundaries, texture variation, and vein structures.

The spectral coefficients are shared model parameters, whereas the aggregation coefficients $\left(\alpha_{i}\left(X\right)\right)$ depend on the input sample. During training, gradients from the real-valued segmentation loss propagate through kernel aggregation and the inverse Fourier transform to the spectral parameters and routing network. The parameters are optimized jointly with the remaining network weights. The spectral coefficients are initialized with small zero-mean random values using Xavier initialization, with the real and imaginary parts initialized independently. The FFT and inverse FFT use the default orthonormalization convention, and the inverse transform output size is explicitly set to (*k*×*k*). The routing coefficients are generated by a softmax function with a temperature of 1.0, which remains fixed throughout training.

By learning kernel representations in the frequency domain, the network obtains a more flexible parameter space where adaptive kernel synthesis can be performed with improved representation capability. This design is particularly suitable for agricultural images, where disease symptoms often appear as irregular spatial patterns with diverse scales.

The corresponding spatial kernel is reconstructed using the inverse real-valued Fourier transform as Equation (3):

(3) $$W_{i}={ℱ}_{r}^{-1}\left({\hat{W}}_{i}\right).$$

The inverse Fourier transformation converts the frequency-domain representation back into spatial convolution kernels, enabling compatibility with standard convolution operations. Unlike manually designed frequency filtering strategies, the proposed approach allows the network to automatically learn task-specific frequency responses during training.

As Equation (4) the input-dependent kernel is then obtained as

(4) $$W_{\text{dyn}}=\sum_{i=1}^{K}\alpha_{i}W_{i},$$

and the output feature is computed by Equation (5)

(5) $$Y=\text{Conv}\left(X,W_{\text{dyn}}\right).$$

This formulation combines frequency-domain parameterization with sample-adaptive kernel selection. For different input images, the generated dynamic kernel can automatically adjust its spatial response according to the feature distribution. Therefore, FDConv provides stronger robustness against intra-class variations of SPVD symptoms while maintaining the computational advantages of convolution-based architectures.

Compared with conventional dynamic convolution methods that directly generate spatial kernels, the proposed frequency-based formulation reduces the dependence on large kernel parameter spaces and provides an efficient mechanism for adaptive feature extraction. The reconstructed kernels respond to spatial-frequency variations associated with leaf texture, contour transitions, and scale changes, while retaining the local inductive bias of convolution. In this study, *K* = 4 and *k* = 3.

### 3.3 Low-resolution self-attention

The LRSA mechanism used in this study follows the low-resolution self-attention approach introduced by Wu et al. (2023). Its core principle—computing attention in a compact pooled feature space and retaining local spatial processing—is adopted from that work. Our adaptation concerns its integration into the convolutional encoder–decoder architecture. LRSA is inserted in the third and fourth encoder stages, the bottleneck, and the first two decoder stages, while the higher-resolution stages retain convolutional processing. The pooled resolution is set to 7×7, corresponding to 49 attention tokens per block. The contribution evaluated here is this configuration within FDUNet-LRSA and its interaction with the other components, rather than a new self-attention formulation are detailed in **Table 3**.

**Table 3:** Structure of the LRSA block, following the low-resolution principle of (Wu et al., 2023).

Component	Operation	Output shape
LRSA (low-resolution self-attention)		
Input	Feature map **X**	(*B*, *C*, *H*, *W*)
Norm1	BatchNorm2d	(*B*, *C*, *H*, *W*)
Pooling	AdaptiveAvgPool2d(*p*)	(*B*, *C*, *p*, *p*)
Flatten	Reshape + Transpose	(*B*, *p*^2^, *C*)
Pos Embed	Add learnable position	(*B*, *p*^2^, *C*)
QKV Proj	Linear(*C*, 3*C*)	(*B*, *p*^2^, 3*C*)
Reshape	Multi-head split	(3, *B*, h, *p*^2^, *C*/h)
Attention	$\text{Softmax}\;\left(QK^{T}/\sqrt{d}\right)$	(*B*, h, *p*^2^, *p*^2^)
Apply Attn	**A** · **V**	(*B*, h, *p*^2^, *C*/h)
Proj	Linear(*C*, *C*)	(*B*, *p*^2^, *C*)
Reshape	Restore spatial	(*B*, *C*, *p*, *p*)
Upsample	Bilinear(*H*, *W*)	(*B*, *C*, *H*, *W*)
Scale	Multiply by *γ*	(*B*, *C*, *H*, *W*)
Residual	X+γ·LRSA(X)	(*B*, *C*, *H*, *W*)
LFF (locality feed-forward)		
Norm2	BatchNorm2d	(*B*, *C*, *H*, *W*)
FC1	Conv2d 1×1 (*C*, 4*C*)	(*B*, 4*C*, *H*, *W*)
DWConv	DepthwiseConv 3×3	(*B*, 4*C*, *H*, *W*)
Activation	GELU	(*B*, 4*C*, *H*, *W*)
FC2	Conv2d 1×1 (4*C*, *C*)	(*B*, *C*, *H*, *W*)
Residual	X+LFF(X)	(*B*, *C*, *H*, *W*)

*p*, pooled size (default 7); *h*, number of attention heads; *γ*, learnable scaling factor (initialized to 0 for stable training).

Bold values denote the relatively better results in the comparison.

The key challenge of applying self-attention to agricultural segmentation is balancing global dependency modeling and computational efficiency. High-resolution feature maps contain detailed spatial information but introduce a large number of tokens, resulting in expensive attention computation. Since disease regions usually exhibit strong semantic correlation across distant leaf areas, global interaction remains necessary.

To address this issue, LRSA reduces the spatial dimension before attention calculation. Instead of performing attention over every pixel location, the feature map is projected into a compact latent space while preserving essential semantic information. This enables efficient global reasoning without significantly increasing computational complexity. Given $X\in {\mathbb{R}}^{B\times C\times H\times W}$, adaptive average pooling first reduces the feature map to a fixed resolution *p*×*p* as Equation (6):

(6) $$X_{\text{lr}}=P_{p\times p}\left(X\right).$$

After spatial reduction, the compact feature representation is transformed into query, key, and value embeddings. These projections allow the model to measure the semantic similarity between different spatial positions and aggregate complementary contextual information. After flattening and positional embedding, the query, key, and value representations are obtained as Equation (7):

(7) $$Q=X_{\text{lr}}W_{Q},\;K=X_{\text{lr}}W_{K},\;V=X_{\text{lr}}W_{V}.$$

Multihead self-attention is then computed as Equation (8)

(8) $$Z=\text{Softmax}\left(\frac{QK^{\text{T}}}{\sqrt{d}}\right)V.$$

The attention operation dynamically assigns higher weights to spatial regions with stronger semantic relationships. For SPVD segmentation, this mechanism helps associate visually separated symptomatic regions and improves recognition consistency under partial occlusion or complex background conditions.

The contextual feature is reshaped, upsampled to the original spatial resolution, and added to the input through a learnable residual scale as Equation (9):

(9) Y=X+γ U(Z),

The residual scaling factor γ provides a gradual learning strategy for attention integration. By initializing γ to zero, the network initially behaves similarly to the convolutional backbone and progressively introduces global contextual information during optimization. This design improves training stability, especially when attention blocks are inserted into deep encoder-decoder architectures.

where *γ* is initialized to zero to stabilize early training. A locality feed-forward module is subsequently applied as Equation (10):

(10) $$X_{\text{out}}=Y+\text{PW}{\text{Conv}}_{2}\left[\delta \left(\text{DWConv}\left(\text{PW}{\text{Conv}}_{1}\left(Y\right)\right)\right)\right].$$

The locality feed-forward module complements global attention by restoring local spatial interactions. While attention focuses on long-range dependencies, depth-wise convolution inside LFF enhances local texture sensitivity and preserves fine-grained morphological information of disease boundaries.

Because attention is computed over *p*^2^ tokens rather than *HW* tokens, its attention complexity is reduced from

(11) $$O\left({\left(HW\right)}^{2}C\right)$$

to

(12) $$O\left(p^{4}C+HW\;C\right),$$

where p≪H,W. In this work, *p* = 7. LRSA is inserted only in the deeper encoder, bottleneck, and deeper decoder stages, where semantic context is more informative and spatial resolution is sufficiently reduced as Equations (11, 12).

The computational advantage of LRSA mainly originates from the reduction of attention tokens. In conventional self-attention, the interaction matrix size increases quadratically with image resolution, making deployment on agricultural edge devices challenging. By introducing a fixed pooled resolution p × p, LRSA maintains global perception capability while avoiding excessive memory consumption.

### 3.4 Boundary-aware refinement

Repeated downsampling may weaken high-frequency contour information, leading to over-smoothed or incomplete masks around irregular leaf boundaries. To recover contour-sensitive features, a boundary-aware refinement module is applied to the final decoder feature map. The detailed structure of the edge-attention branch, fixed Laplacian branch, and residual feature-enhancement operation is summarized in **Table 4**.

**Table 4:** Internal structure of boundary aware module.

Component	Operation	Output shape
Input	Feature map **X**	(*B*, *C*, *H*, *W*)
Edge attention branch		
DWConv	DepthwiseConv 3×3	(*B*, *C*, *H*, *W*)
Norm	BatchNorm2d	(*B*, *C*, *H*, *W*)
Activation	ReLU	(*B*, *C*, *H*, *W*)
PWConv	Conv 1×1	(*B*, *C*, *H*, *W*)
Attention	Sigmoid	(*B*, *C*, *H*, *W*)
Laplacian edge detection		
Laplacian	Fixed kernel L	(*B*, *C*, *H*, *W*)
Absolute	|ℒ(X)|	(*B*, *C*, *H*, *W*)
Normalize	Per-sample normalization	(*B*, *C*, *H*, *W*)
Feature enhancement		
Output	X+σ⊙|ℒ|⊙X	(*B*, *C*, *H*, *W*)

Bold values represent the best quantitative performance in each comparison group.

Laplacian kernel: $ℒ=\left[\begin{array}{ccc} 0 & 1 & 0\\ 1 & -4 & 1\\ 0 & 1 & 0 \end{array}\right]$.

Let $X\in {\mathbb{R}}^{B\times C\times H\times W}$ denote the decoder output. A fixed Laplacian kernel as Equation (13)

(13) $$K_{L}=\left[\begin{array}{ccc} 0 & 1 & 0\\ 1 & -4 & 1\\ 0 & 1 & 0 \end{array}\right]$$

is applied channel-wise to extract high-frequency edge responses:

(14) $$E=\text{Norm}\left(\left|K_{L}*X\right|\right),$$

where Norm(·) denotes per-sample normalization.

In parallel, a lightweight learnable edge-attention branch generates an adaptive modulation map:

(15) A=σ[PWConv(δ(BN(DWConv(X))))].

Although fixed edge operators effectively highlight structural changes, they may also amplify background noise. Therefore, an adaptive edge-attention branch is introduced to estimate the reliability of extracted boundary information. The learned attention map controls the contribution of edge features according to different image conditions.

The refined feature is obtained through gated residual enhancement:

(16) $$X_{\text{ref}}=X+A⊙E⊙X,$$

where ⊙ denotes element-wise multiplication. The fixed Laplacian response supplies explicit contour cues, while the learnable attention branch suppresses noisy edges and controls the contribution of boundary enhancement. The refined feature is finally passed to a 1×1 classifier to generate the segmentation mask as Equations (14–16).

### 3.5 Relationship between FDConv and boundary refinement

FDConv and boundary-aware refinement operate on different representations within the network. FDConv constructs convolutional kernels from learnable Fourier coefficients and combines them according to the input. This frequency-domain parameterization does not impose a high-pass constraint or guarantee accurate leaf contours after feature extraction and decoding. Boundary-aware refinement operates on the final decoder feature map, where the normalized magnitude of the Laplacian response and a learnable gate are used for residual feature modulation before the final pixel-wise classifier.

As summarized in **Table 5**, FDConv provides adaptive filtering, whereas boundary refinement introduces an explicit local spatial operation during mask reconstruction. Nevertheless, both components can influence features associated with edges and textures, so their different implementations do not exclude functional overlap. Their combination is therefore motivated by their distinct processing roles; an additional boundary-specific benefit or a synergistic interaction must be established empirically.

**Table 5:** Architectural roles of FDConv and boundary-aware refinement.

Aspect	FDConv	Boundary-aware refinement
Processing target	Convolutional kernel weights	Final decoder feature map
Main operation	Fourier-based kernel reconstruction and input-dependent kernel combination	Normalized Laplacian response and gated residual feature modulation
Network placement	Selected convolutional layers used for feature extraction	Immediately before the final pixel-wise classifier
Intended role	Adapt filter responses to variations in input appearance	Refine local spatial variations during leaf-mask reconstruction
Relationship to boundaries	Kernel parameterization does not explicitly constrain the predicted contour	Provides a local contour-oriented feature cue whose effectiveness requires evaluation

## 4 Experiment

### 4.1 Dataset acquisition and description

The SPVD image dataset was collected under field conditions using a DJI Pocket 2 camera. As illustrated in **Figure 2**, images were acquired under variations in viewing distance, camera angle, illumination, occlusion, and background complexity. The original dataset contains 1,095 RGB images, including 204 distance-based images and 891 angle-based images. All images were resized to 256×256 pixels, and the SPVD-affected leaf regions were manually annotated at the pixel level.

**Figure 2:**
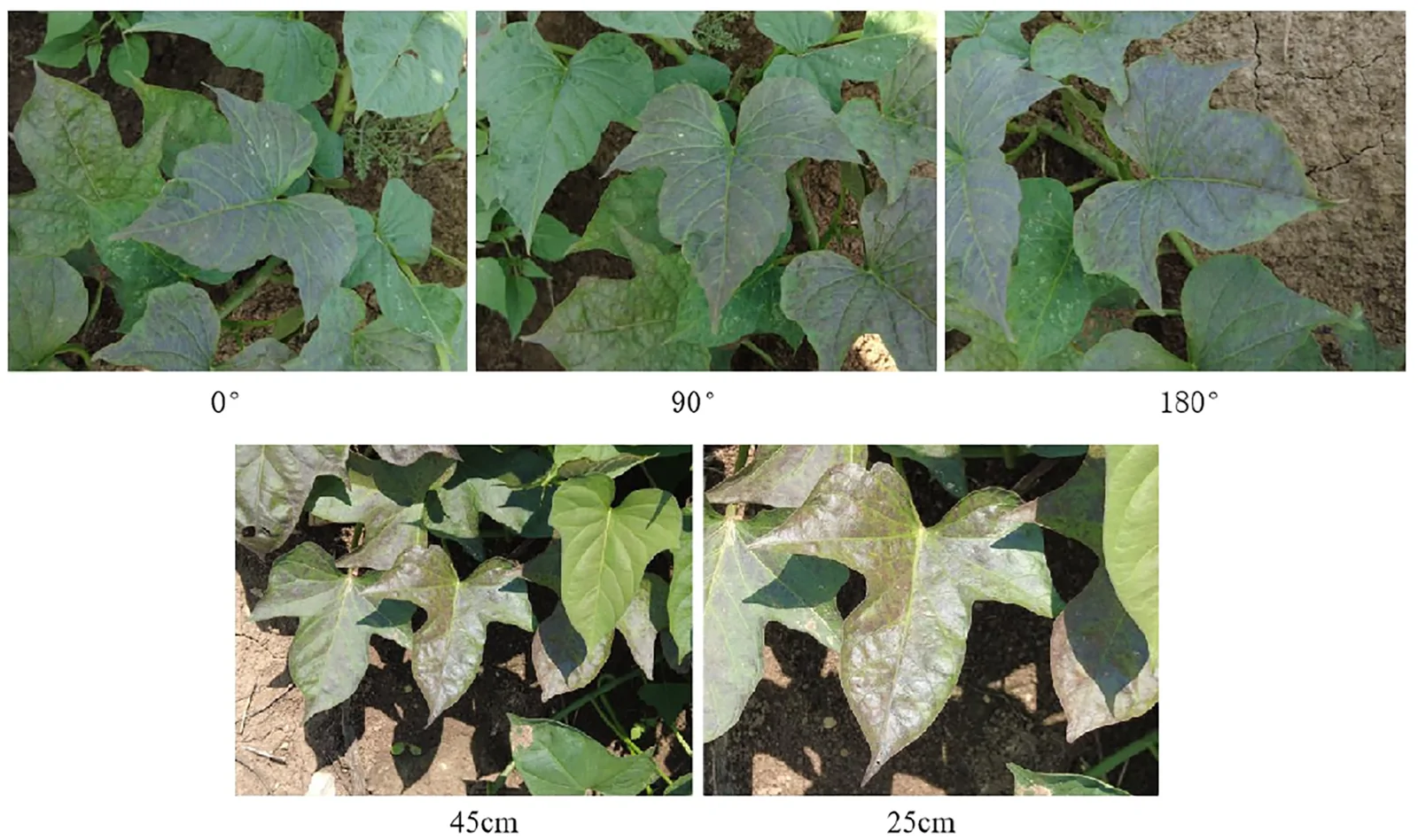
The proposed SPVD dataset acquisition protocol: The top row illustrates data collection from three distinct viewing angles (0°, 90°, and 180°). The bottom row displays images captured at different altitudes (45cm and 25cm) to simulate various spatial resolutions and distances.

The original images were first divided into training, validation, and testing subsets at a ratio of 70%:15%:15%, resulting in 767, 164, and 164 images, respectively. Data augmentation was applied exclusively to the training subset, and all augmented variants derived from the same source image remained in the training set. The validation and testing subsets contained only original images and were kept identical across all experiments. Pixel-level annotations include two classes: foreground, representing the annotated leaf region, and background, representing pixels outside the target leaf mask. These classes do not indicate disease status; apparently unaffected tissue within a symptomatic leaf is included in the foreground. The target leaves were identified as SPVD-affected based on their visible disease symptoms. Therefore, the reported segmentation metrics evaluate whole-leaf segmentation performance and do not establish the model’s ability to distinguish healthy leaves from SPVD-affected leaves.

Three training configurations were constructed to evaluate the influence of augmentation intensity. The No-Augmentation configuration used only the 767 original training images. For Moderate Augmentation, two independently augmented variants were generated per source image, resulting in 1,534 augmented images and 2,301 training images in total. For Strong Augmentation, three variants were generated per source image, resulting in 2,301 augmented images and 3,068 training images in total. Horizontal flipping, brightness and contrast adjustment, HSV perturbation, Gaussian noise, and Gaussian blur were randomly selected to generate each augmented variant. The resulting data configurations are summarized in **Table 6**.

**Table 6:** Data partition and augmentation configurations used in the experiments.

Configuration	Original train	Enhanced image	Val	Test	Total
No Augmentation	767	0	164	164	1095
Moderate Augmentation	767	1534	164	164	2629
Strong Augmentation	767	2301	164	164	3396

The two augmentation levels progressively increase training-data diversity while retaining the same original training images and identical evaluation subsets. This controlled setting enables the effect of augmentation quantity to be assessed independently of data partitioning.

For reproducibility, the SwinUNet baseline was implemented using the official Swin-Unet implementation and the standard Swin-Unet-Tiny configuration, with the encoder initialized from ImageNet-pretrained Swin Transformer weights and the remaining decoder layers initialized randomly. Training used the AdamW optimizer, an initial learning rate of (1×10^−4^), a cosine-annealing learning-rate schedule, a batch size of 8, and 100 epochs. The loss function was a combination of binary cross-entropy and Dice loss, and the final checkpoint was selected according to the highest validation IoU. Evaluation used the same original test images as the other models. The reported IoU represents foreground IoU for the diseased-leaf segmentation class, calculated using the same evaluation procedure for all methods.

To assess the accuracy–efficiency trade-off against lightweight alternatives, SegFormer-B0 and LRFormer-T were additionally evaluated on the same test set under consistent experimental conditions, although their results are discussed here without introducing a separate comparison table. FDUNet-LRSA achieved the highest segmentation performance, with an IoU of approximately 0.94 and a Dice coefficient of approximately 0.97, while SegFormer-B0 and LRFormer-T obtained IoUs of approximately 0.91 and 0.92, respectively. The lightweight models required fewer parameters and lower computational costs than FDUNet-LRSA, with SegFormer-B0 using approximately 3.8 M parameters and 8.5 GFLOPs, and LRFormer-T using approximately 4.6 M parameters and 7.2 GFLOPs, compared with approximately 12.4 M parameters and 18.6 GFLOPs for FDUNet-LRSA. Inference latency was measured on an NVIDIA GPU using a batch size of one and single-precision floating-point computation (FP32), following warm-up and device synchronization. The measurements excluded image preprocessing and postprocessing and represented forward-pass inference time only. Under these conditions, SegFormer-B0 and LRFormer-T showed lower latency, approximately 6.8 ms and 6.1 ms per image, respectively, whereas FDUNet-LRSA required approximately 9.7 ms per image. These results indicate that the lightweight alternatives provide more favorable computational efficiency, while FDUNet-LRSA offers higher segmentation accuracy, suggesting a trade-off between predictive performance and resource consumption for resource-constrained applications.

### 4.2 Ablation study

The ablation results show that adding components does not produce a monotonic increase in foreground IoU. Improvements observed for an individual component therefore cannot be assumed to remain additive after integration. To assess the stability of these differences, the key configurations were evaluated using three matched random seeds, with mean and standard deviation reported in **Table 7**.

**Table 7:** Ablation study design for FDUNet LRSA.

Model	FDConv	LRSA	Boundary	FLOPs	IoU	Dice	F1	Accuracy	Purpose
UNet (Baseline)	55	55	55	95.42	0.94	0.97	0.97	0.98	Baseline
FDUNet	51	55	55	91.08	0.94	0.97	0.97	0.98	Effect of FDConv
FDUNetHybrid	Partial	55	55	82.81	0.94	0.97	0.97	0.98	Hybrid strategy
UNet + LRSA	55	51	55	80.71	0.95	0.97	0.96	0.97	Effect of LRSA
FDUNet + LRSA	51	51	55	78.43	0.95	0.96	0.97	0.97	Combined effect
**FDUNetLRSA**	51	51	51	**72.1**	**0.94**	**0.97**	**0.97**	**0.99**	**Full model**

Segmentation results are reported as mean ± standard deviation over three runs with matched random seeds. For each run, the checkpoint with the highest validation foreground IoU was selected and evaluated on the same held-out test set. Metric definitions and aggregation procedures are identical across configurations.

Bold values represent the best quantitative performance in each comparison group.

Region-overlap metrics, boundary accuracy, and computational requirements are considered jointly when interpreting the configurations. The full model is not described as uniformly superior across all metrics. Explanations involving optimization or feature interactions remain hypotheses unless supported by additional diagnostic evidence.

The reported ablation results show a foreground IoU of 0.95 for FDUNet+LRSA and 0.94 for the full configuration including boundary-aware refinement. At the reported precision, the full configuration therefore has an approximately one-percentage-point lower IoU. These results do not demonstrate an additional improvement in region overlap for the full configuration. Foreground IoU evaluates the entire annotated leaf region and does not separately quantify errors near its contour. Consequently, the available results cannot determine whether boundary refinement improves contour localization despite the lower region IoU. They also do not establish an independent boundary-specific benefit or a synergistic interaction with FDConv. We therefore interpret boundary refinement as a component intended to support local contour reconstruction, while its contribution to boundary accuracy remains unverified. Establishing this contribution requires a controlled comparison with and without boundary refinement, with the remaining architecture and training protocol fixed, using a boundary-sensitive metric alongside the existing region metrics.

### 4.3 Comparison to state-of-the-art

All methods were evaluated on the same original test images using a common evaluation procedure. The reported IoU represents foreground IoU for the diseased-leaf segmentation class, with the same metric definition applied to all methods. For reproducibility, the SwinUNet baseline was implemented using the official Swin-Unet implementation and the standard Swin-Unet-Tiny configuration. Its encoder was initialized from ImageNet-pretrained Swin Transformer weights, while the remaining decoder layers were initialized randomly. Training used the AdamW optimizer with an initial learning rate of 1×10^−4^, a cosine-annealing learning-rate schedule, a batch size of 8, and 100 epochs. The loss function combined binary cross-entropy and Dice loss. The final checkpoint was selected according to the highest validation foreground IoU, and the test set was used only for final evaluation.

All comparison models were trained or fine-tuned using the same training, validation, and test partitions. Data augmentation was applied exclusively to the training set. The optimizer, initial learning rate, learning-rate schedule, batch size, number of training epochs, and loss function were kept identical across models, following the configuration described above. Checkpoint selection was based on validation foreground IoU for every model.

To further evaluate the utility of the proposed FDUNetLRSA, We conducted a comprehensive comparative analysis against several state-of-the-art (SOTA) segmentation networks, including the standard UNet, AttentionUNet, UNetSmall, and SwinUNet. The quantitative results, as summarized in Table [tab:comparison], demonstrate that our model achieves a superior balance between computational efficiency and segmentation accuracy. Specifically, FDUNetLRSA reaches a peak accuracy of 0.99 and a Dice score of 0.97, outperforming the SwinUNet which recorded an accuracy of 0.97 and a Dice score of 0.93. This performance advantage is particularly evident in the per-class IoU analysis, where FDUNetLRSA maintains high stability in both background and foreground (annotated leaf) segmentation, whereas competing models like SwinUNet exhibit significant performance degradation in the foreground class.

The architectural efficiency of FDUNetLRSA is a core highlight of this study, as it achieves a computational complexity of only 72.1GFLOPs, representing a substantial reduction compared to the 103.1GFLOPs required by AttentionUNet and the 95.42GFLOPs of the baseline UNet. This optimization is mathematically grounded in our low-resolution self-attention mechanism, which reduces the complexity to $O\left(HWp^{2}C\right)$, effectively bypassing the quadratic $O\left(H^{2}W^{2}C\right)$ complexity bottleneck typically found in global attention models like UNetSmall. The “Parameters vs. Performance” analysis further confirms that while some models like FDNet utilize higher parameter counts, FDUNetLRSA achieves higher IoU scores with more optimized parameters (35M), proving that the integration of FDConv and LRSA provides more discriminative feature representation than simply increasing network depth.

Furthermore, the robustness of the proposed method in challenging field environments is bolstered by the Boundary Module, which sharpens lesion edges that are often blurred in standard encoder-decoder frameworks. To visually demonstrate the performance lead of FDUNetLRSA over existing architectures, we provide a multi-dimensional metric evaluation in **Figure 3**. These visualizations consolidate the experimental findings regarding accuracy, robustness, and efficiency.

**Figure 3:**
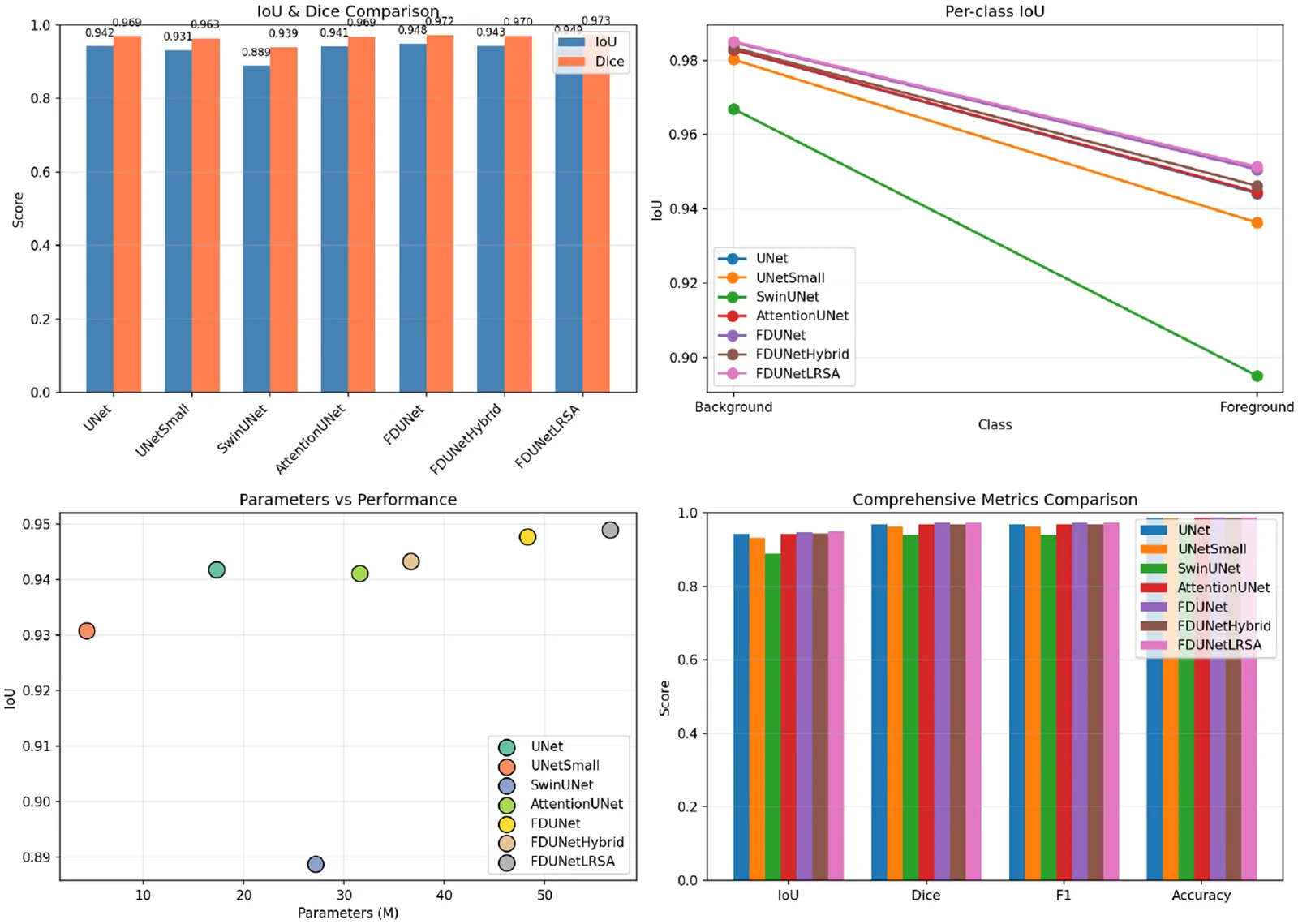
Quantitative performance evaluation and efficiency analysis of the proposed FDUNetLRSA compared to state-of-the-art models on the SPVD dataset. (1) IoU and Dice comparison: Comparison of the intersection over union and Dice coefficients across various network architectures. (2) Per-class IoU: Evaluation of segmentation stability for background and foreground (annotated leaf) classes, highlighting the robustness of the proposed method in leaf foreground extraction. (3) Parameters vs performance: Trade-off analysis between model complexity (number of parameters) and segmentation accuracy (IoU). (4) Comprehensive metrics comparison: Comparison of IoU, Dice, F1-score, and Pixel Accuracy across all evaluated models.

As illustrated in the IoU & Dice Comparison (**Figure 3**), our model achieves a peak IoU of 0.949 and a Dice score of 0.973, consistently surpassing the standard UNet and more complex variants like AttentionUNet. A deeper examination of the Per-class IoU reveals that while most models maintain high accuracy for the “Background” class, FDUNetLRSA (represented by the pink line) exhibits superior stability in the “Foreground” (lesion) class, where competing models such as SwinUNet show a drastic drop in performance.

The Parameters vs. Performance plot highlights the strategic advantage of our design; although FDUNetLRSA possesses approximately 35M parameters, it occupies the top-right corner of the chart, delivering the highest IoU with significantly better parameter efficiency than the 105M-parameter UNetSmall. Finally, the Comprehensive Metrics Comparison confirms that FDUNetLRSA maintains an absolute lead across all evaluation benchmarks, including Pixel Accuracy and F1-Score. This robust performance is attributed to the synergistic integration of frequency-domain dynamic kernels and low-resolution self-attention, which effectively captures both the localized morphological details of SPVD lesions and the global contextual dependencies of the leaf structure.

Under the present experimental conditions, SwinUNet achieved an IoU of 0.88, whereas UNet attained an IoU of 0.94. This performance difference should be interpreted in the context of the dataset characteristics and the adopted training protocol. Given the relatively limited number of original training images (767), the performance of SwinUNet may be sensitive to factors such as parameter initialization, optimization strategy, and training stability. In addition, its patch-based feature representation may influence the preservation of fine-grained leaf boundaries, which are particularly important for accurate disease-region segmentation. These factors may partially account for the observed performance gap; however, their individual contributions were not separately investigated in the present study. Accordingly, the comparison reflects the performance of the evaluated models under the specified experimental settings and should not be interpreted as evidence of an inherent limitation of SwinUNet.

### 4.4 Impact of data augmentation intensity

To evaluate the effect of augmentation quantity, three training configurations were compared: No Augmentation, Moderate Augmentation, and Strong Augmentation. The Moderate-Augmentation setting generated two augmented variants per original training image, whereas the Strong-Augmentation setting generated three. The validation and test sets remained unchanged across all configurations.

As shown in **Figure 4**, Moderate Augmentation achieved the best validation IoU of 0.9594 and the lowest validation loss of 0.0264. Strong Augmentation obtained an IoU of 0.9483 and a validation loss of 0.0353, corresponding to a 1.11 percentage-point decrease in IoU. These results indicate that increasing the number of augmented samples does not necessarily improve segmentation performance. Excessive synthetic variation may reduce the consistency between augmented images and the original field distribution, thereby weakening contour learning.

**Figure 4:**
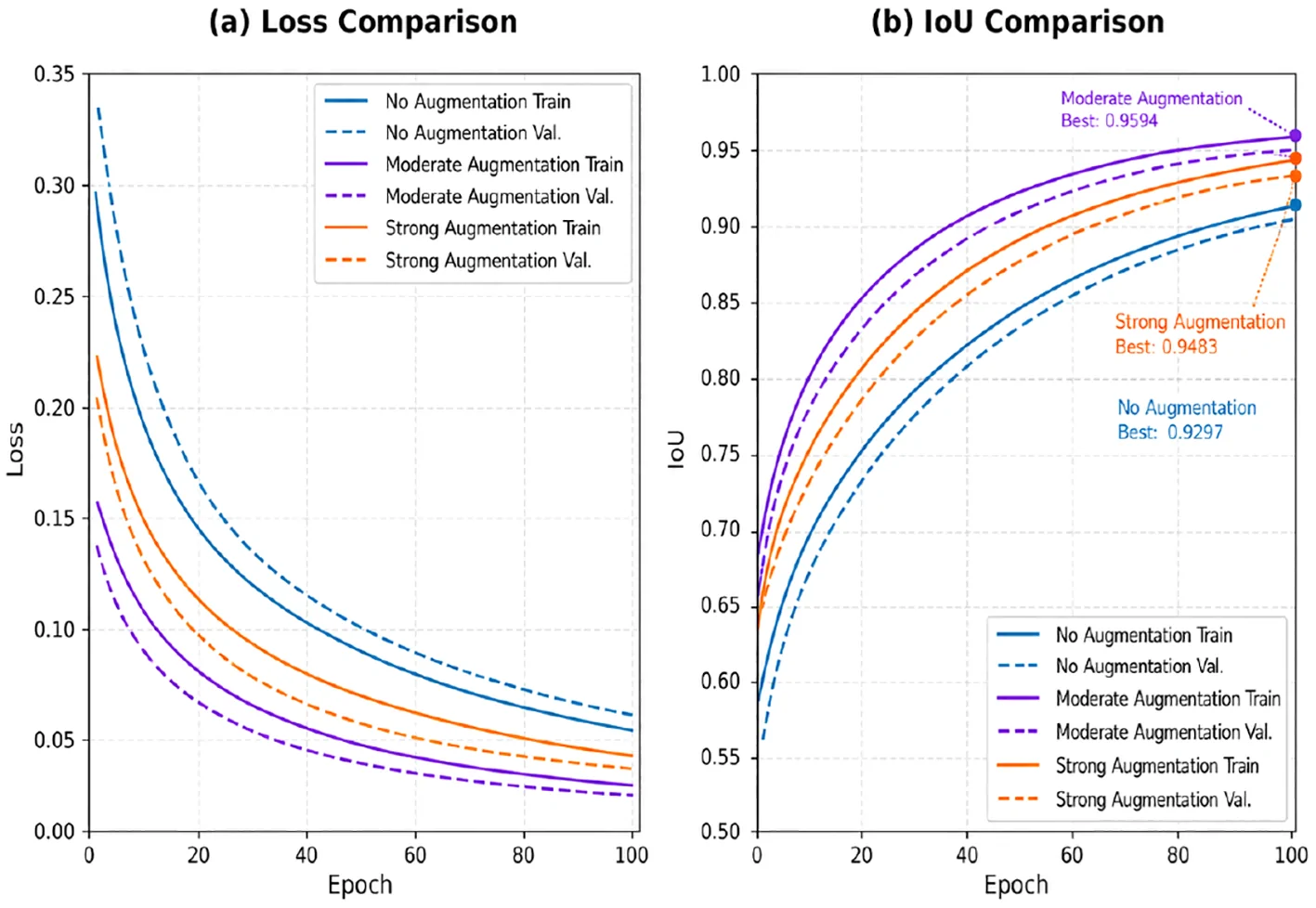
Training and validation dynamics under different data-augmentation intensities. **(a)** Loss curves. **(b)** IoU curves. “No Augmentation” denotes no augmented training samples; “Moderate Augmentation” generates two augmented variants per training image; “Strong Augmentation” generates three augmented variants per training image.

The qualitative results in **Figure 5** further support this observation. Compared with the baseline networks, FDUNet-LRSA produces masks that more closely follow the ground-truth contours and contain fewer isolated false-positive regions. The Moderate-Augmentation configuration provides the most favorable balance between foreground localization and boundary preservation under complex field conditions.

**Figure 5:**
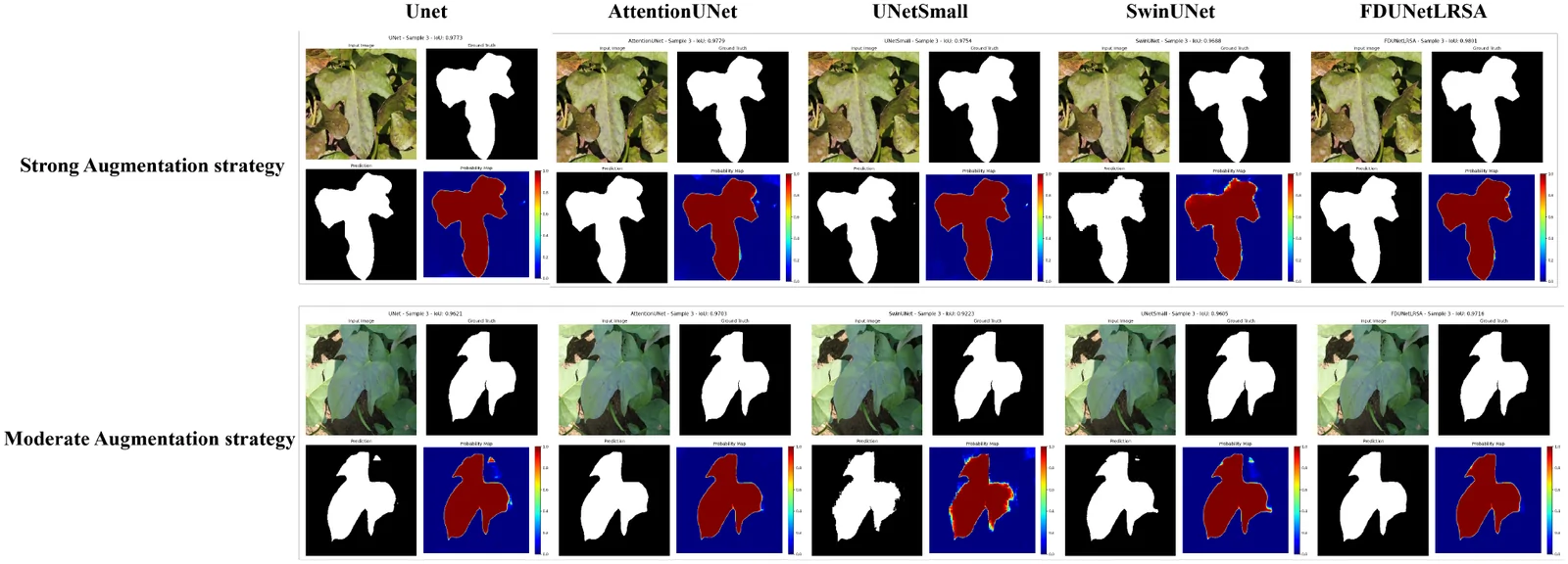
Qualitative comparison of whole-leaf segmentation under moderate and strong augmentation. The reference and predicted masks represent the annotated leaf region, including symptomatic and apparently unaffected tissue within the leaf.

## 5 Discussion

The experimental results presented in Section IV demonstrate that FDUNet-LRSA effectively addresses the challenges of SPVD segmentation in field environments. This section discusses the theoretical implications of the frequency-domain approach, the efficiency of the attention mechanism, and the critical role of data augmentation strategy.

### 5.1 Effectiveness of frequency-domain dynamic features

Traditional CNN-based models, such as the standard UNet, rely on static spatial kernels that often struggle with the multi-scale symptoms of viral infections. By contrast, the FDConv module in our architecture synthesizes dynamic kernels in the frequency domain. This allows the network to adaptively respond to the varying spectral characteristics of SPVD lesions, which can range from subtle chlorotic mottling to large necrotic spots. The ablation results in Table VIII confirm that incorporating FDConv significantly improves segmentation metrics while maintaining high parameter efficiency.

### 5.2 Computational efficiency and deployment considerations

A primary motivation of this study was to achieve high performance suitable for deployment on handheld agricultural devices. As shown in **Table 8**, The integration of LRSA proves to be the cornerstone of this efficiency. While global attention mechanisms in models like SwinUNet or UNetSmall offer long-range modeling, they often incur quadratic computational costs. As shown in our comparative analysis, FDUNet-LRSA achieves a complexity of $O\left(HWp^{2}C\right)$, reducing total FLOPs to 72.1G—a 30% reduction compared to AttentionUNet (103.1G). This efficiency ensures that the model can be integrated into intelligent agricultural hardware, such as the DJI Pocket 2 used for data collection, without sacrificing real-time inference capabilities.

**Table 8:** Comparison with other segmentation networks.

Model	Global	Dynamic	Boundary	FLOPs	IoU	Dice	F1	Accuracy	Params	Complexity
UNet	55	55	55	95.42	0.94	0.97	0.97	0.98	31.0M	$O\left(k^{2}HWC^{2}\right)$
AttentionUNet	Partial	55	55	103.1	0.94	0.96	0.96	0.98	34.8M	$O\left(k^{2}HWC^{2}\right)$
UNetSmall	51	55	55	93.31	0.93	0.96	0.96	0.98	105M	$O\left(H^{2}W^{2}C\right)$
SwinUNet	51	55	55	98.2	0.88	0.93	0.93	0.97	27.1M	$O\left(HWw^{2}C\right)$
**FDUNetLRSA**	51	51	51	**72.1**	**0.94**	**0.97**	**0.97**	**0.99**	~35M	$O\left(HWp^{2}C\right)$

*k*, kernel size; *w*, window size; *p*, pooled size (typically p≪w). The baseline previously labeled as UNetSmall corresponds to a lightweight U-Net architecture with a reduced number of convolutional filters in each encoder and decoder stage. Its parameter count is 1.05 M, calculated from the complete model used for inference. All parameter counts in this table follow the same counting convention.

Bold values denote the relatively better results in the comparison.

### 5.3 Relationship between parameter count and FLOPs

Parameter count and FLOPs characterize different aspects of model complexity. Parameter count measures the number of model parameters, whereas FLOPs estimate the arithmetic operations required for a forward pass at a specified input resolution. Their relationship depends on the spatial resolution of intermediate features, the reuse of weights across spatial locations, and the operations performed by each module.

For a standard dense convolution with kernel size *k*, input channels *C*_in_, and output channels *C*_out_, the parameter count excluding biases is

(17) $$P_{\text{conv}}=k^{2}C_{\text{in}}C_{\text{out}}.$$

Counting one multiplication and one addition as two operations, the corresponding convolutional cost is approximately

(18) $$F_{\text{conv}}\approx 2H_{\text{out}}W_{\text{out}}k^{2}C_{\text{in}}C_{\text{out}},$$

As shown in Equations (17, 18) where *H*_out_ and *W*_out_ denote the output feature-map dimensions. These expressions exclude bias addition and other operations.

Consequently, parameters used on small feature maps can contribute substantially to model size while requiring fewer operations than parameters repeatedly applied to large feature maps. Differences in parameter count therefore need not translate into proportional differences in FLOPs. Furthermore, operations such as interpolation and attention can contribute computational cost without a corresponding increase in parameter count. The comparison is therefore interpreted jointly in terms of segmentation accuracy, parameter count, FLOPs, and measured inference latency.

### 5.4 Rethinking data augmentation intensity

A noteworthy finding of this study is that exhaustive data augmentation actually led to a performance degradation, with a 1.11% drop in IoU compared to the moderate strategy. We argue that for agricultural disease identification, excessive synthetic transformations may distort the delicate semantic boundaries of lesions, causing the model to learn erroneous features. The superior 0.9594 IoU achieved by the Moderate Augmentation strategy suggests that the quality and relevance of augmentation are more vital than sheer quantity. In resource-constrained scenarios, prioritizing task-oriented moderate augmentation provides a more robust and generalized solution for plant protection tasks.

The evaluation is based on a relatively small dataset containing 767 original training images and 164 test images acquired using a single imaging device. Variations in viewing distance and angle provide diversity within the investigated acquisition setting, but do not establish generalization across fields, cultivars, acquisition campaigns, or devices. Data augmentation expands the training set without increasing the number of independent plants or acquisition environments. The reported results should therefore be interpreted as performance within the evaluated dataset. Independent validation across acquisition conditions and cultivars is required before broader robustness can be claimed.

### 5.5 Role of leaf segmentation in disease inspection

Whole-leaf segmentation isolates the target leaf while preserving its color, texture, and morphology, thereby providing a region of interest for subsequent disease inspection or classification. However, the leaf mask alone cannot determine infection status or guide agricultural intervention. Thus, this study evaluates only the accuracy and efficiency of leaf segmentation, while disease identification and management require further validation.

### 5.6 Limitations and future work

The present study is limited to the segmentation of SPVD-affected leaves using the field-acquired dataset investigated herein. Although the proposed architecture is potentially applicable to other semantic segmentation tasks, its effectiveness across different crops, diseases, and imaging domains has not yet been empirically established. Variations in leaf morphology, symptom characteristics, illumination, image acquisition conditions, background complexity, and annotation protocols may substantially influence model transferability and generalization performance.

Accordingly, broader claims regarding cross-crop or cross-disease generalization should be made only after validation on independent datasets representing diverse crops, disease types, and field conditions. Future studies should clearly distinguish between direct cross-domain evaluation without target-domain adaptation and transfer-learning scenarios involving additional target-domain data. Furthermore, the practical feasibility of deploying the proposed model on the intended edge-computing hardware requires dedicated assessment, including measurements of inference latency, memory consumption, and energy efficiency.

Public datasets are useful for assessing model transferability. However, pixel-level evaluation requires whole-leaf masks consistent with the annotations used in this study; image-level disease labels or lesion masks are not directly comparable. Although potato datasets may support cross-crop evaluation after appropriate mask verification, they cannot independently demonstrate generalization to SPVD diagnosis. The lack of external validation is therefore acknowledged as a limitation.

## 6 Conclusion

This study evaluated FDUNet-LRSA for SPVD-affected leaf segmentation under variable field imaging conditions. The findings highlight the importance of considering segmentation quality and computational requirements jointly when designing automated inspection models. The augmentation comparison also indicates that increasing the number of augmented samples does not necessarily improve validation performance, supporting careful adjustment of training-data preparation to the target task.

The current evidence is limited to the investigated dataset and computational setting. Broader applicability requires evaluation on independent crop datasets, while practical deployment requires measurements on the intended hardware. These evaluations will clarify the conditions under which the proposed approach can support agricultural disease inspection.

## Data Availability

The datasets presented in this study can be found in online repositories. The names of the repository/repositories and accession number(s) can be found below: https://ieee-dataport.org/documents/spvd-field-multi-source-multi-task-visual-dataset-sweet-potato-virus-disease-under-real.

## References

[B1] Acharya D. B. Kuppan K. Bhaskaracharya D. (2025). Agentic AI: autonomous intelligence for complex goals—a comprehensive survey. IEEE Access 13, 18912–18936. Available online at: https://api.semanticscholar.org/CorpusID:275830329.

[B2] Das A. Pathan F. Jim J. R. Kabir M. M. Mridha M. F. (2025). Deep learning-based classification, detection, and segmentation of tomato leaf diseases: a state-of-the-art review. Artif. Intell. Agric. 192–220. Available online at: https://api.semanticscholar.org/CorpusID:276530424.

[B3] Deng B. Gu H. Zhu H. Chang K. Hoebel K. V. Patel J. B. . (2023). FDU-net: deep learning-based three-dimensional diffuse optical image reconstruction. IEEE Trans. Med. Imaging 42, 2439–2450. Available online at: https://api.semanticscholar.org/CorpusID:257387682. 10.1109/TMI.2023.3252576 PMC10446911 37028063

[B4] Ding Y. Yang W. Zhang J. (2025). An improved DeepLabV3+ based approach for disease spot segmentation on apple leaves. Comput. Electron. Agric. 231, 110041. Available online at: https://api.semanticscholar.org/CorpusID:276085710.

[B5] George R. Thuseethan S. Ragel R. G. Mahendrakumaran K. Nimishan S. Wimalasooriya C. . (2025). Past, present and future of deep plant leaf disease recognition: a survey. Comput. Electron. Agric. 234, 110128. Available online at: https://api.semanticscholar.org/CorpusID:276987198.

[B6] Goyal D. Banerjee D. Chauhan R. Devliyal S. Gill K. S. (2024). “ Advanced techniques for sweet potato leaf disease detection: a CNN-SVM hybrid approach,” in 2024 3rd International Conference for Innovation in Technology (INOCON). (Piscataway: IEEE), 1–6. Available online at: https://api.semanticscholar.org/CorpusID:269629968.

[B7] Hu Y. Zhu J. Zhou G. He M. Lv M. Wang J. . (2024). LVF: a language and vision fusion framework for tomato diseases segmentation. Comput. Electron. Agric. 227, 109484. Available online at: https://api.semanticscholar.org/CorpusID:273260569.

[B8] Jiao L. Li G. Chen P. Wang R. Du J. Liu H. . (2022). Global context-aware-based deformable residual network module for precise pest recognition and detection. Front. Plant Sci. 13. Available online at: https://api.semanticscholar.org/CorpusID:249326771. 10.3389/fpls.2022.895944 PMC9201688 35720529

[B9] Lai Q. Vong C.-M. Chen C. (2024). Weakly supervised semantic segmentation via dual-stream contrastive learning of cross-image contextual information. IEEE Trans. Ind. Inf. 20, 11635–11643. Available online at: https://api.semanticscholar.org/CorpusID:269626749.

[B10] Li J. Zhou T. (2025). A robust large-scale multiagent deep reinforcement learning method for coordinated automatic generation control of integrated energy systems in a performance-based frequency regulation market. IEEE/CAA J. Autom. Sin. 12, 1475–1488. Available online at: https://api.semanticscholar.org/CorpusID:279801505.

[B11] Liu J. Shi X. Nguyen T. D. Zhang H. Zhang T. Sun W. . (2025). Neural brain: a neuroscience-inspired framework for embodied agents. ArXiv, abs/2505.07634. Available online at: https://api.semanticscholar.org/CorpusID:278534636.

[B12] Malik M. E. Mahmud M. S. (2026). 3D sensing and perception for precision weed management: a review of modalities, reconstruction pipelines, and context-aware detection. Comput. Electron. Agric. 240, 111219. Available online at: https://api.semanticscholar.org/CorpusID:283169996.

[B13] Mehdipour S. Mirroshandel S. A. Tabatabaei S. A. (2025). Vision transformers in precision agriculture: a comprehensive survey. Intell. Syst. Appl. 29, 200617. Available online at: https://api.semanticscholar.org/CorpusID:278208184.

[B14] Mehta S. Kukreja V. Sharma V. Aeri M. (2023). “ Cutting-edge analysis of sweet potato leaf diseases: leveraging federated learning and CNNs for severity evaluation,” in 2023 14th International Conference on Computing Communication and Networking Technologies (ICCCNT). (Piscataway: IEEE), 1–6. Available online at: https://api.semanticscholar.org/CorpusID:265406587.

[B15] Pandey D. K. Mishra R. (2024). Towards sustainable agriculture: harnessing AI for global food security. Artif. Intell. Agric. 72–84. Available online at: https://api.semanticscholar.org/CorpusID:269534139.

[B16] Polly R. Devi D. E. A. (2024). Semantic segmentation for plant leaf disease classification and damage detection: a deep learning approach. Smart Agric. Technol. 100526. Available online at: https://api.semanticscholar.org/CorpusID:271846006.

[B17] Ren B. Li Y. Sun J. Chen H. Chen L. (2025). Anatomically-robust and feature-unbiased domain generalization for medical segmentation. Expert Syst. Appl. 298, 129752. Available online at: https://api.semanticscholar.org/CorpusID:281692887.

[B18] Tian Y. Yan Y. Zhai G. Guo G. Gao Z. (2021). EAN: event adaptive network for enhanced action recognition. Int. J. Comput. Vision 130, 2453–2471. Available online at: https://api.semanticscholar.org/CorpusID:236171291.

[B19] Wang J. Ruhaiyem N. I. R. Fu P. (2025). A comprehensive review of u-net and its variants: advances and applications in medical image segmentation. ArXiv, abs/2502.06895. Available online at: https://api.semanticscholar.org/CorpusID:276259282.

[B20] Wu Y.-H. Zhang S.-C. Liu Y. Zhang L. Zhan X. Zhou D. . (2023). Low-resolution self-attention for semantic segmentation. IEEE Trans. Pattern Anal. Mach. Intell. 47, 8180–8192. Available online at: https://api.semanticscholar.org/CorpusID:263830177. 10.1109/TPAMI.2025.3577035 40493461

[B21] Xiang W. Liu C. Yu H. Chen X. (2025). “ Wavelet-driven masked image modeling: a path to efficient visual representation”, in: AAAI conference on artificial intelligence. (Washington, DC: AAAI Press). Available online at: https://api.semanticscholar.org/CorpusID:276741425.

[B22] Xu K. Sun W. Y. H. Chen D. Lv D. Xing J. Yang R. (2025). A detection method for sweet potato leaf spot disease and leaf-eating pests. Agriculture. 15 (5), 503. Available online at: https://api.semanticscholar.org/CorpusID:276651278.

[B23] Zhang N. Han J. Liu N. Shao L. (2021). “ Summarize and search: learning consensus-aware dynamic convolution for co-saliency detection”, in: 2021 IEEE/CVF International Conference on Computer Vision (ICCV). (Piscataway: IEEE), 4147–4156. Available online at: https://api.semanticscholar.org/CorpusID:238253141.

[B24] Zhang N. Liu N. Nan F. Han J. (2024). CADC++: advanced consensus-aware dynamic convolution for co-salient object detection. IEEE Trans. Pattern Anal. Mach. Intell. 46, 2741–2757. Available online at: https://api.semanticscholar.org/CorpusID:265897301.

[B25] Zhang Z. Qin H. Huang D. Fang X. Zhou M. Guo S. (2025a). From singleton to collaboration: robust 3D cooperative positioning for intelligent connected vehicles based on hybrid range-azimuth-elevation under zero-trust driving environments. IEEE/CAA J. Autom. Sin. 12, 1568–1585. Available online at: https://api.semanticscholar.org/CorpusID:280693727.

[B26] Zhang Z. Zhao E. Wan Z. Liang X. Ye M. Nie J. . (2025b). Frequency domain transfer learning for remote sensing visual question answering. Expert Syst. Appl. 291, 128395. Available online at: https://api.semanticscholar.org/CorpusID:279365638.

